# Mechanisms by which hydrogen sulfide attenuates muscle function following ischemia–reperfusion injury: effects on Akt signaling, mitochondrial function, and apoptosis

**DOI:** 10.1186/s12967-018-1753-7

**Published:** 2019-01-21

**Authors:** Michael D. Wetzel, Joseph C. Wenke

**Affiliations:** US Army Institute of Surgical Research, Extremity Trauma and Regenerative Medicine, 3698 Chambers Pass BLDG 3611, Ft. Sam Houston, San Antonio, TX 78234 USA

**Keywords:** Hydrogen sulfide, Muscle, Ischemia reperfusion injury, Apoptosis, Akt, Mitochondria, eNOS

## Abstract

Ischemia–reperfusion injury is caused by a period of ischemia followed by massive blood flow into a tissue that had experienced restricted blood flow. The severity of the injury is dependent on the time the tissue was restricted from blood flow, becoming more severe after longer ischemia times. This can lead to many complications such as tissue necrosis, cellular apoptosis, inflammation, metabolic and mitochondrial dysfunction, and even organ failure. One of the emerging therapies to combat ischemic reperfusion injury complications is hydrogen sulfide, which is a gasotransmitter that diffuses across cell membranes to exert effects on various signaling pathways regulating cell survival such as Akt, mitochondrial activity, and apoptosis. Although commonly thought of as a toxic gas, low concentrations of hydrogen sulfide have been shown to be beneficial in promoting tissue survival post-ischemia, and modulate a wide variety of cellular responses. This review will detail the mechanisms of hydrogen sulfide in affecting the Akt signaling pathway, mitochondrial function, and apoptosis, particularly in regards to ischemic reperfusion injury in muscle tissue. It will conclude with potential clinical applications of hydrogen sulfide, combinations with other therapies, and perspectives for future studies.

## Background

Ischemic-reperfusion injury (IR) occurs when there is a restriction of blood flow to tissue, followed by massive reperfusion caused by sudden blood flow to the affected area. Deprived of oxygen cells rely on anaerobic metabolism during IR, resulting in decreases in pH, followed by reduction of available ATP and calcium overload in cells. This is accompanied by opening of the mitochondrial permeability transition pore (mPTP), disrupting mitochondrial membrane potential and electron transport chain [1]. Lack of oxygen can also lead to capillary dysfunction and breakdown of cell membranes, contributing to tissue necrosis [1–3]. IR can affect many tissues, including brain, intestine, kidney, heart, and skeletal muscle. It is also associated with impaired healing of chronic wounds, organ transplant complications, and tourniquet application [3–5]. IR can be a result of different types of injuries that include compartment syndrome, crush injuries, and vascular injuries [3]. In addition to loss of blood flow and nutrients to affected tissues IR is exasperated by increased inflammation and reactive oxygen species (ROS) release, which cause further damage to cells and can initiate apoptosis by mPTP opening and caspase activation [3, 6, 7].

Muscle, particularly skeletal muscle, is one of the primary tissues affected by IR, which is marked by changes in microvasculature, muscle volume, loss of function, and increased inflammation [3, 8, 9]. Different tissues have specific critical times before onset of serious injury; for muscle this is approximately 4 h [8]. Beyond this time unrepairable tissue necrosis and tissue loss occurs due to mitochondrial loss and apoptotic activation, which can necessitate amputation of the affected limb [10–12]. Different types of muscle display differing response to ischemia based on their mitochondrial content. Highly oxidative muscles such as the soleus displayed less severe damage in response to IR than glycolytic muscles such as the gastrocnemius, likely due to increased anti-oxidant presence in oxidative muscles [9]. Additionally, IR can affect organs beyond the affected limb by increases of inflammatory cytokines. For example, kidney and heart cells are extremely vulnerable to restrictions of blood flow, and introduction of free radical scavengers can improve total body function in ischemic animal models by reduction of inflammatory cytokines such as interleukins (IL) and tumor necrosis factor alpha (TNFα) [13–19].

Hydrogen sulfide (H_2_S) is a gasotransmitter, along with nitric oxide (NO) and carbon monoxide (CO) that initiates a variety of signaling pathways within cells. Hydrogen sulfide has traditionally been thought of as a poisonous gas emitting a rotten egg smell, but recent evidence suggests that in micromolar amounts H_2_S can alter various signaling pathways involved in vasodilation, metabolism, apoptosis, and mitochondrial electron transport chain (ETC) [20–23]. In addition to environmental H_2_S that is absorbed across cell membranes via diffusion cells are also able to produce small amounts of endogenous H_2_S by reverse transsulfuration of dietary L-homocysteine [24]. This process is mainly carried out by the cytosolic enzymes cystathionine β-synthase (CBS; mostly found in nerves) and cystathionine γ-lyase (CSE; mostly found in muscle), which utilize cystathione to convert homocysteine to cysteine, with H_2_S as a by-product [24, 25]. Additionally, H_2_S can also be generated by mitochondrial mercaptopyruvate sulphur transferase (3-MST), which utilizes mercaptopyruvate to form a persulfide intermediate by cysteine transanimation of α-ketoglutarate and l-cysteine. Presence of a reducing agent such as thioredoxin then releases H_2_S and pyruvate [25, 26]. Once released from cells H_2_S has a short half-life of up to 12 min in vivo (in contrast to aerosol half-life of up to 37 h), making continuous endogenous production of H_2_S critical to its activity [27, 28]. Interestingly, it has been shown that the three major endogenous hydrogen sulfide producing enzymes (CBS, CSE, 3-MST), as well as total hydrogen sulfide are reduced in muscle and kidney following ischemia, which can be attenuated through introduction of H_2_S donors [29–31], suggesting that H_2_S can be useful in reducing IR complications. Once released H_2_S can modify proteins by sulfurhydration to augment preservation by cryoprotection, alter ion channel activity (K^+^, Ca^2+^, K_ATP_), regulate apoptosis by affecting Akt (also known as protein kinase B) and phosphoinosiol kinase (PI3K)-mammalian target of rapamycin (mTOR), reduce inflammation, act as a free radical scavenger, and alter mitochondrial electron transport chain activity by alteration of K_ATP_ pore formation and regulation of cyclic AMP (cAMP) activity [21, 24, 26, 32–34]. H_2_S uses the K_ATP_ pump as a second messenger system, and is also proposed to cross-talk with the other gasotransmitters by regulation of endothelial nitric oxide synthase (eNOS) and heme oxygenase-1 to affect the activities of NO and CO, respectively [26, 35]. H_2_S can be introduced in vivo by both fast release (sodium hydrosulfide: NaHS) and slow release Morpholin-4-ium 4 methoxyphenyl (morpholino) phosphinodithioate: (GYY4137), diallyl trisulfide, with differing effects. The fast release donors release their drug in a quick burst which are effective for short-term effects such as immediate modulation of cell metabolism and inflammation reduction, while the slow release donors are more effective at promoting long-term effects on muscle recovery and tissue integrity due to gradual release of H_2_S [36, 37].

Hydrogen sulfide has been proposed as a therapy to prevent IR damage by reducing free radical induced stress, promoting mitochondrial function, activating vascularization pathways, and reducing apoptosis. Administration of H_2_S donors have improved survival following myocardial infarct in mice when combined with cardiopulmonary resuscitation [38]. Most research has been focused on the effects of H_2_S as a therapeutic agent in cardiac ischemia, with little knowledge on its effects in skeletal muscle. This review will cover the effects of H_2_S in reducing IR mostly in cardiac muscle, with some inferences drawn from studies in other tissues. The primary focus will be on metabolic effects associated with IR injury, primarily Akt-eNOS signaling, mitochondrial ETC and mPTP activity, and cellular apoptosis.

## H_2_S ameliorates detrimental effects of muscle ischemia by altering Akt signaling

Binding of insulin growth factor 1 (IGF-1) to its receptor activates a signaling cascade that activates PI3K to activate downstream Akt and adenosine monophosphate kinase (AMPK) [33, 39, 40]. Akt functions in muscle synthesis by promoting protein synthesis by activating mammalian target of rapamycin (mTOR), and preventing degradation by inactivating forkhead box (FoxO) gene transcription [33, 40]. One possible mechanism of preventing muscle damage following IR is to activate Akt to promote mTOR activation by phosphorylation of Akt at Ser473 and Thr308. One study showed that hearts isolated from rats pretreated with 50 µM NaHS prior to IR displayed increased Akt and mTOR phosphorylation, along with decreased cell death and improved coronary flow [33]. This same study also showed that H9c2 cardiomyocyte cells treated with the mTOR inhibitor PP242 prevented NAHS induced mTOR phosphorylation and cardioprotection [33]. Timing and dosage of NaHS are also critical, as 100 µM NaHS decreased heart function, and administration following reperfusion did not result in increased mTOR phosphorylation in isolated heart tissue [33]. A report which pretreated H9c2 cells with 100 µM NaHS for 30 min prior to inducing cardiotoxicity with 5 µM doxorubicin, and found that NaHS pretreatment increased Akt and FoxO3a phosphorylation, which was decreased by doxorubicin alone [32]. The action of H_2_S was augmented by the free radical scavenger N-acetyl-l-cysteine (NAC). Disruption of Akt activity by the inhibitor LY294002 prevented Akt anti-apoptotic activity, and resulted in increased nuclear localization of FoxO3a [32], demonstrating that Akt activity is indispensable for H_2_S protection of cardiomyocytes against cell death. Additionally, mTOR complex 2 can promote Akt1 phosphorylation, which promotes cardioprotection that is ablated by the mTOR inhibitor PP242, even in the presence of exogenous 50 µM NaHS [33]. H_2_S can also exert cardioprotective functions when administered post-ischemia. Yong et al. [41] performed a study measuring cardiac function and metabolic activation following 100 µM NaHS administered post-myocardial infarct in either six-ten second administrations or a single 2 min administration. It was observed that NaHS administration, particularly in the six-ten second intervals, resulted in reduced infarct size, with increased phosphorylation of Akt and protein kinase C [41]. Blockage of Akt or PKC with 15 µM LY294002 or 10 µM chelerythrine resulted in reduced NaHS induced cardioprotection in the 2 min continuous administration model [41], suggesting that timing of H_2_S donor administration can activate different pathways. These data suggest that the Akt-mTOR pathway is a critical regulator of muscle function and survival in ischemia, and the absence of either is detrimental to muscular function. The protective effects of Akt activation by H_2_S are not limited to muscle; it has been demonstrated that an increase in Akt phosphorylation by NaHS pretreatment in hepatic IR, which was abolished by inhibiting Akt with LY294002 [42], suggesting that Akt is a pathway that can be targeted in many tissues affected by IR. Taken together, this data suggests that activation of the Akt pathway can reduce ischemia in various organs by attenuating mTOR induced cell proliferation, and Akt activity is essential for H_2_S activity in all tissues.

## Effects of H_2_S and Akt on vascularization

Akt also crosstalks with NO signaling pathways to promote vascularization through vascular endothelial growth factor (VEGF) activation [35, 43]. Deficiencies of either H_2_S or NO levels have been linked with increased risk of cerebral IR by vascular restriction [44]. VEGF is a potent pro-angiogenic factor that promotes vascularization in ischemia and cancer through a variety of signaling pathways such as Akt and STAT3 [32, 40, 45, 46]. In ischemic muscle addition of VEGF can result in reduced damage and improved function. Rats subjected to hind limb ischemia induced by unilateral external iliac and femoral artery and vein ligation, then injected with an alginate gel containing 3 µg of VEGF and/or IGF-1 demonstrated that either treatment resulted in improved vascularization measured by laser Doppler perfusion injury, reduced fibrosis, and improved muscle regeneration and function [47]. VEGF and IGF-1 acted in synergy to improve ischemia response superior to either treatment alone [47]. Administration of H_2_S donor once or twice a day using a dose of .25–.05 mg/kg over 7 days resulted in increased blood flow to rat hind limbs following femoral artery ligation [48]. Another study involved implanting muscle derived stem cells into mice with muscular dystrophy [49]. It was found that while stem cells alone stimulated in vivo angiogenesis and muscle regeneration, responses were improved when the cells were transduced to overexpress VEGF. Cells expressing soluble forms-like tyrosine kinase-1 displayed significantly less vascularization and increased fibrosis [49], demonstrating that VEGF is crucial to re-establishment of vascularization following IR. In addition to blood flow, VEGF also promotes innervation of damaged muscles. Introduction of VEGF containing gel into damaged human sternomastoid displayed 50 percent innervated motorend plates, compared to only 5 percent for blank gels [47, 50]. VEGF administration also increased expression of nerve growth factor and glial-derived neurotrophic factor, improving axonal regeneration in damaged muscle. Inhibition of the nerve growth factors disrupted VEGF induced nerve repair [50], showing that VEGF acts on a variety of vascular, neural, and cell growth signaling pathways to repair muscular damage and restore function. VEGF activity is augmented by eNOS, and VEGF also acts to upregulate eNOS expression in endothelial cells, forming a feedback loop [51, 52]. Akt is involved in the eNOS-VEGF signaling pathway as an upstream regulator, and has been implicated in regulation of vascularization in many ischemic tissues [35, 51, 53]. One experiment induced hind limb ischemia in rats by femoral artery ligation, followed by daily intraperitoneal NaHS injection (50 µmol/kg). It was found that Akt, VEGF, and VEGF receptor 2 activity all increased in endothelial cells near the ligation site, along with increased measured vascular flow [54], demonstrating a role for Akt in VEGF induction. The study by Yong et al. showed that the 10 s administrations of NaHS resulted in increased expression of eNOS, suggesting that eNOS was critical to NaHS induction of angiogenesis [41]. H_2_S administration reduced cardiac failure induced by transverse aortic constriction by upregulation of eNOS that was dependent on CSE activity, as CSE knockout mice did not respond to H_2_S donor administration [55]. A similar study found that a large dose (100 µg/kg) of Na_2_S restored eNOS activity in CSE knockout mice, but mice lacking phospho eNOS activity were unresponsive to H_2_S donor treatment following IR [56]. eNOS is critical to angiogenesis during ischemia, as eNOS knockout resulted in absence of vascularization following NaHS administration [35]. Dietary H_2_S sources can also induce Akt and eNOS induced vascularization, as demonstrated by a study done injecting mice with daillyl trisulfide, a component of garlic oil that contains H_2_S. A daily injection of 500 µg/kg daillyl trisulfide over 10 days resulted in improved blood flow following hind limb ischemia, along with increased Akt and eNOS phosphorylation. Akt and eNOS knockout mice were not affected by the treatment, demonstrating that both are essential to vascular repair in IR [57]. It has been shown that dietary and environmental sources (ozone, garlic, vitamin E) have been effective in promoting IR healing through free radical scavenging, and likely also vascular signaling [13, 16, 20, 57–59]. The effects of H_2_S in improving IR response through Akt-eNOS are not limited to muscle, as demonstrated by use of H_2_S donors to improve angiogenesis following ischemia in intestine [35] and brain [53], suggesting a wide range of targets for H_2_S directed therapies.

In addition to affecting vascularization through VEGF and eNOS, H_2_S can also directly interact with NO to produce nitroxyl, which has a longer half-life than either NO or H_2_S. Both H2S and NO presence are neccesary to produce nitroxyl, and they work synergestically to promote smooth muscle relaxation and portal vein vasodilation [60]. Low nitroxyl concentrations have been shown to be beneficial to vasodilation, cardiac function, smooth muscle relaxation, and cGMP activity, although high levels can be neurotoxic and inflammatory [60, 61]. More research is necessary to deduce the exact roles of H_2_S and NO crosstalk on VEGF activity and vascularization in order to safely induce vascularization while avoiding adverse side effects.

## Summary of the effects of H_2_S on Akt activity and IR

**Fig. 1:**
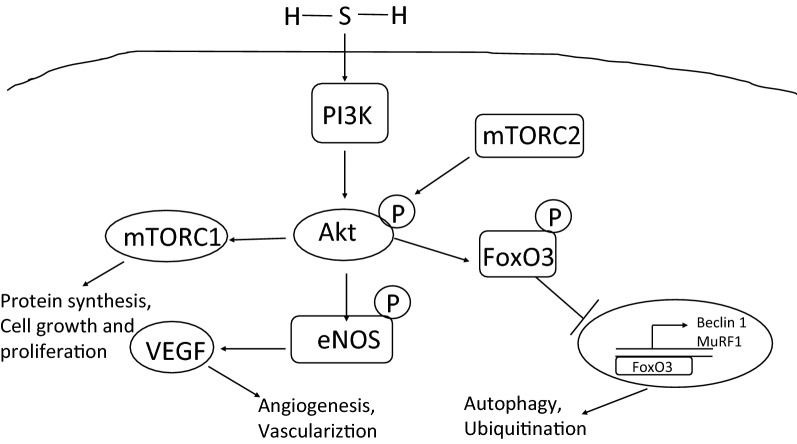
Roles of H_2_S in modulating Akt signaling pathways involved in ischemia reperfusion injury and recovery. H_2_S activates PI3 K and downstream Akt phosphorylation, which can also be activated by mTORC2. Akt can subsequently activate mTORC1 to regulate protein synthesis and cell proliferation, eNOS to activate vascularization by VEGF, and induce phosphorylation of FoxO_3_ to prevent activation of genes promoting autophagy and ubiquitination

The mechanism of H_2_S to regulate Akt pathways is shown in Fig. 1. H_2_S activates PI3 K, which then phosphorylates Akt. Once activated Akt can activate eNOS, which is upregulated by VEGF and VEGF receptor. VEGF also functions synergistically with eNOS to promote vascular function, which is disrupted if either eNOS or Akt is blocked. Akt activates the mTOR complex to promote protein synthesis and cell growth. Akt also phosphorylates FoxO3 to prevent its translocation to the nucleus, preventing autophagy via Beclin 1 and protein degradation via activation of the ubiquitin proteosome pathway by MuRF1 [62]. Akt can also be activated by mTOR complex 2 to augment its activity [33]. Akt is a pluripotent signaling regulator that regulates many pathways that regulate cell survival and angiogenesis, making it a prime target for IR therapeutic strategies.

## Mitochondrial electron transport chain function

The mitochondrial electron transport chain (ETC) is comprised of 5 subunits that utilize the electron carriers nicotinamide adenine dinucleotide (NADH) and flavin adenine dinucleotide (FADH_2_) to ultimately drive ATP production via a proton gradient. Electrons flow through the ETC along the five complexes embedded in the mitochondrial inner membrane. Complex I oxidizes NADH and reduces coenzyme Q. Succinate is oxidized by complex II, which also reduces CoQ. Complex III oxidizes coenzyme Q and reduces cytochrome C that donates an electron to complex IV, reducing oxygen to water. Complex V couples proton flow from the outer to the inner membrane with an electrochemical gradient to drive the complex to phosphorylate ADP. The activity of complex V is regulated by ADP levels; in low activity states the rate of ATP hydrolysis is reduced, resulting in less available ADP to be phosphorylated by complex V [63]. Respiration uncoupling occurs in pathologic states due to damage to the inner membrane or to complex V, resulting in loss of ADP regulation, impaired energy production, and increased mitochondrial permeability [63].

## Effects of mitochondrial ETC dysfunction in IR

During IR there is an alteration of metabolic pathways such as AMPK, which is a regulator of mitochondrial activity through activation of liver kinase B1. AMPK can be activated by stress associated with IR, leading to decreases in available cellular ATP [15, 64]. One of the major features of IR is a shift towards anaerobic metabolism, as demonstrated by decreases in blood glucose and increases in lactate, glycerol, and pyruvate measured by microdialysis in the early stages of reperfusion [5, 65]. During ischemia lack of available oxygen results in increased pyruvate and cellular acidification. Cells attempt to remove the excess acid by use of the Na^+^/H^+^ and Na^+^/Ca^2+^ pumps, which leads to increased cytosolic Ca^2+^ levels. The inhibition of Ca^2+^ transport by low ATPase activity is a result of decreased ATP production by the oxygen dependent electron transport chain [66]. Acidic cellular pH and NADH accumulation keep the mPTP closed during ischemia, but during reperfusion the rapid resumption of oxidative phosphorylation and restoration of normal cytosolic pH results in mitochondrial Ca^2+^ uptake, which opens the mPTP, resulting in free radical release and cell death [66, 67]. Surviving cells are affected by ROS, which cause mitochondrial proton leak and uncoupling of the electron transport chain from ATP pump activity, exasperating the available ATP crisis seen in IR. There is conflicting evidence on the extent of mitochondrial dysfunction in IR. One study showed that a 1 h tourniquet application did not result in significant changes in citrate synthase or mitochondrial complex I-III activities [11]. However another study showed that while a 25 min cardiac ischemia followed by 3 min of reperfusion did not change mitochondrial complex enzymatic activity, it did result in complex I thiol modifications and increased ROS production [68], suggesting that even short IR results in increases in oxidative stress within mitochondria. It has been shown that cardiac IR resulted in decreased complex III activity, lipid peroxidation, and hydrogen peroxide production, which were attenuated by hypoxic but not normoxic reperfusion [7], so the rapid introduction of oxygen following ischemia results in superoxide and free radical production that result in tissue damage and mitochondrial dysfunction. A more severe ischemic injury, such as dual hind limb artery ligation mimicking peripheral artery disease resulted in decreased respiration across mitochondrial complexes I, III, and IV, along with increased magnesium superoxide dismutase (MnSOD) expression [69], suggesting that the length and severity of the IR is a major factor in preserving mitochondrial oxidative function. It is imperative to find therapeutic methods to reduce the severity of mitochondrial damage in IR, which has been linked with detrimental outcomes in ischemic stroke due to alterations in mitochondrial metabolic intermediates such as NADH and acetyl CoA, along with mPTP associated stress [70].

## H_2_S mediated attenuation of IR induced mPTP activation

Hydrogen sulfide has been implicated in protection against myocardial infarction by modulation of mitochondrial activity, particularly in regulating the mPTP. Activation of the K_ATP_ channel by protein kinase C inhibits opening of the mPTP, which prevents ROS release and cell death [71]. Treating cardiomyocytes with NaHS resulted in increased PKC translocation to the cell membrane, increased mitochondrial membrane potential, and elevated K_ATP_ channel activation, resulting in decreased mitochondrial cytochrome C release, less mPTP activation, and improved cardiomyocyte survival [72]. H9c2 cardiac cells treated with 400 µM NaHS and the K_ATP_ channel openers diazoxide or pinacidil were protected from high glucose induced stress by increasing K_ATP_ activity and reducing oxidative stress [73]. Inhibition of mPTP and cytochrome C release by NaHS treatment has also been observed in rat lungs subjected to acute lung injury, along with reduced mitochondrial swelling and improved lung pathology [74]. Seven day administration of NaHS (5.6 mg/kg/day) in a mouse model of Parkinson’s disease resulted in decreased neuronal cell death and increased K_ATP_ channel activity [75]. It has even been demonstrated that inhaled H_2_S had neuroprotective effects in mice exposed to neurotoxin 1-methyl-4-phenyl-1,2,3,6-tetrahydropyridine (MPTP) to induce neural damage [76]. The mechanism by which H_2_S affects K_ATP_ activity is under debate. The K_ATP_ channel is a heterooctomeric complex that is encoded by the Kir6.1 and Kir6.2 genes that encode the pore forming subunits, and the sulfonylurea receptor (SUR)1 and SUR2 regulatory subunits [71]. The genes for Kir6.1 and SUR2 are next to each other on chromosome 12p12.1, as are Kir6.2 and SUR1 on chromosome 11p15.1, suggesting that the subunits are under similar regulatory control [77, 78]. In muscle the Kir6.1 and SUR2 subunits are predominately expressed [71]. Colonic smooth muscle cells treated with 1 mM NaHS displayed increased K_ATP_ activity and SUR2 but not Kir6.1 sulfhydration [79], suggesting that H_2_S can increase expression or at least activity of K_ATP_ pore subunits. One report showed that rats exposed to inhaled tobacco smoke displayed lower levels of serum H_2_S, and reduced gene expression of CSE and SUR2 in aortic smooth muscle, inhibiting both endogenous H_2_S levels and action [80]. However another study observed that NaHS protected against neuronal damage in both wild type and Kir6.2 knockout mice, and that uncoupling protein (UCP) 2 knockout mice were not affected by NaHS administration, leading to the suggestion that H_2_S activity was dependent on the presence of UCP-2, not K_ATP_ pore subunits [75]. More research needs to be done to determine the effects of H_2_S on K_ATP_ pore subunit expression in ischemic muscle.

## H_2_S alteration of mitochondrial cAMP activity

**Fig. 2:**
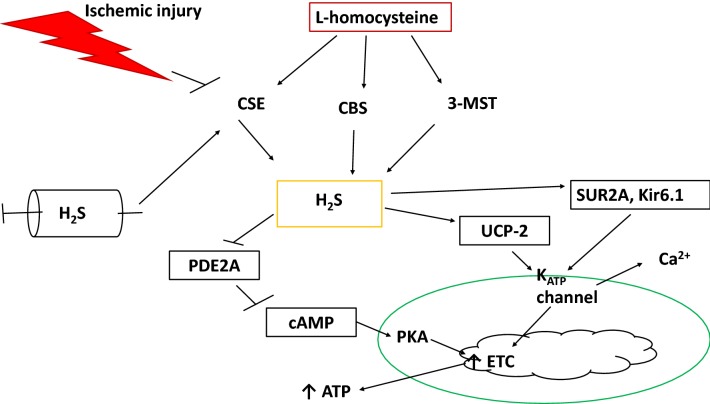
Restortion of mitochondrial ATP synthesis pathways by ischemia and H_2_S in muscle. Dietary L-homocysteine undergoes a reverse transsulfuration reaction by CBS, CSE, and 3-MST to produce endogenous H_2_S. Ischemia reduces expression of these enzymes, resulting in decreased endogenous H_2_S production. Introduction of an exogenous H_2_S donor (NaHS) can increase expression of the enzymes, resulting in increased endogenous H_2_S synthesis. This affects mitochondria by increasing K_ATP_ activity through increased gene expression of SUR2A-Kir6.1 and/or acting through UCP-2. This leads to increased Ca^2+^ export form the mitochondria, stimulating ATP transport. H_2_S also upregulates cAMP by inhibiting PDE2A, resulting in increased mitochondrial PKA activity to stimulate the electron transport chain (ETC), resulting in increased oxygen utilization and ATP production

H_2_S also affects mitochondrial activity through modulation of cyclic AMP (cAMP) activity. cAMP is a second messenger that can exert many effects on muscles, including stimulating glucose transport and activating protein kinase A to stimulate mitochondrial electron transport chain activity [81–83]. It has been shown that livers from rats treated with 10 µM NaHS displayed increased cAMP activity and PKA expression [21]. cAMP is inhibited in the mitochondria by phosphodiesterase 2A (PDE2A), which was inhibited by exogenous NaHS, resulting in increased mitochondrial PKA and electron transport chain activity [21]. This activity has not yet been observed in ischemic muscles, but research into the effects of H_2_S on mitochondrial cAMP-PDE2A activity could determine if H_2_S exerts ubiquitous or cell type specific effects on mitochondrial electron transport chain regulation. Since it has been shown that the presence of exogenous H_2_S stimulates production of the three endogenous H_2_S producing enzymes (CBS, CSE, 3-MST) [29], it is possible that H_2_S treatment could alter mitochondrial activity by increasing endogenous production, and affecting downstream mitochondrial function by increasing activity of the K_ATP_ channel and/or cAMP signaling. These pathways potentially regulating the effects of H_2_S on mitochondrial activity in muscle are illustrated in Fig. 2. IR can inhibit endogenous H_2_S production by CSE, which results in downregulation of K_ATP_ pore subunits and UCP-2. It also prevents ETC stimulation as a result of cAMP inhibition by PDE2A. These effects can be reversed by introduction of exogenous H_2_S, making H_2_S an attractive candidate for attenuating mitochondrial induced cell death by reducing mPTP opening.

## H_2_S and complex IV inhibition

Hydrogen sulfide also acts as a reversible inhibitor of mitochondrial complex IV, the terminal complex in the ETC, thus reducing mitochondrial oxidative phosphorylation [84]. H_2_S has been associated with reduced metabolism by inducing a hibernation like state. Blackstone et al. demonstrated that rats exposed to 80 ppm of inhaled H_2_S for 6 h displayed less oxygen utilization with lower core body temperature and carbon dioxide output [85]. The rats returned to a normal metabolic state after being returned to room air, suggesting that H_2_S induced hypometabolic state is easily reversible and produces no long term detrimental effects [84, 85], thus potentially reducing trauma following injury by lowering mitochondrial metabolic function. Upon return to normal conditions the rats displayed no behavioral detriments that have been associated with long term hydrogen sulfide exposure [85]. Several studies have shown that inhaled hydrogen sulfide up to 100 ppm for 30 min rapidly induces a hypometabolic state that reduces inflammation, apoptosis, and tissue function in IR models of renal and lung damage and hypoxia by reducing oxidative stress and mitochondrial oxygen utilization [86–88]. Interestingly, H_2_S and cryopreservation been used to preserve kidneys for successful transplantations while avoiding IR associated tissue damage that is not attenuated by cryopreservation alone [89]. To date there has been no work done on H_2_S induced preservation of skeletal muscle following IR, opening up an interesting possibility for future investigation.

## H_2_S reduces ischemic necrosis by reducing cellular apoptosis

Hydrogen sulfide has been widely implicated in the prevention of cellular apoptosis under ischemic conditions. One of the major pathways implicated is the PI3 K-Akt signaling pathway. In addition to stimulating mTOR as described previously, Akt has also been implicated in preventing cell death by activating c-Jun-N-terminal kinase (JNK). Rat cardiomyocytes treated with NaHS or the JNK inhibitor SP600125 displayed inhibited phosphorylation of JNK, which led to decreased cytochrome C release, an increase in B-cell lymphoma (Bcl)-2 expression, and increased cell survival [90]. The timing of NaHS treatment was critical, as administration 1 h following reperfusion did not prevent cellular apoptosis [90]. NaHS has been shown to protect primary human umbilical vein endothelial cells from high glucose induced stress by deactivating Bax through upregulation of Bcl-2, deactivated downstream caspase 3, and upregulated superoxide dismutase, reducing apoptosis by 41 percent compared to untreated cells [91]. Akt activation has also been implicated in protecting hippocampal neurons from IR stress by activating glycogen synthase kinase (GSK)3β and glutamate NMDA receptor subunit epsilon (NR)2A and B [92]. The Akt-NR2 pathway in cerebral IR is augmented by heat shock protein (HSP) 70, as induction of Akt by inhaled H_2_S also activates HSP70 to prevent cerebral IR induced neural apoptosis, resulting in increased cognitive function measured by Morris maze test [93]. Retinal ganglion cells from rats treated with inhaled H_2_S prior to retinal IR displayed less apoptosis via regulation of JNK, HSP-90, and caspase-3 [94], suggesting a wide variety of IR types that can be attenuated with H_2_S treatment. Another study also demonstrated that inhaled H_2_S prevented neuronal apoptosis induced by MPTP by upregulation of antioxidant genes [76]. GSK3β activation through Akt has also been implicated in protecting cardiomyocytes from apoptosis by preventing mPTP opening and Bax translocation [95]. The connection between ROS, inflammation, and apoptosis in IR is well established [2, 30, 34, 96–100]. The inflammatory cytokines TNFα and IL-6 are increased in hepatic reperfusion, which can be attenuated by 5 µM NaHS treatment, along with increased Bcl-2 and decreased Bax and JNK activation [99]. Autophagy was also affected, as Beclin-1 and microtubule-associated protein 1A/1B-light chain 3 (LC3) were decreased by NaHS, resulting in improved hepatic structure and decreased autophagasome detection [99]. Two independent studies found that mouse hepatocytes treated with NaHS displayed increased Akt and GSK3β phosphorylation, correlating with decreased Beclin-1 and LC3 [42, 101], suggesting an Akt-JNK-GSK3-Bcl-2-mPTP pathway activated by IR that is modulated by NaHS in preventing apoptosis and autophagy. This pathway might be augmented by HSP70 and 90, which can encode genes involved in ROS scavenging and antioxidants such as thioredoxin-1, and downregulation of inflammatory TNFα, IL-6, and NF-κB signaling [24, 34, 46, 102–104]. Other studies have determined that H_2_S reduces inflammatory cytokine levels in many cell and tissue types, particularly TNFα and IL-6, suggesting a potent anti-apoptotic function for H_2_S by deactivating inflammation induced Bax signaling and subsequent mPTP opening, which prevents against further cell damage [18, 19, 105, 106]. For example one study identified that NaHS prevented IL-6 secretion from PC12 neural cells subjected to hypoxic and glucose deprivation stress, allowing for protection against many sources of inflammation that are induced by IR [107]. H_2_S appears to be multifunctional in affecting many pro-apoptotic pathways associated with increased IR damage, making it an attractive candidate for reducing IR associated cell death and tissue necrosis.

## H_2_S reduction of stress induced apoptosis

As previously mentioned, H_2_S can attenuate inflammatory cytokines and ROS associated with IR. A few studies have determined that H_2_S also protects skeletal muscle from IR induced apoptosis by reducing stress and inflammation. Henderson et al. [108] performed a study delivering 10 µM NaHS 20 min prior to 3 h of tourniquet induced hind limb ischemia, followed by 3 h of reperfusion, and found that H_2_S treated mice showed a reduced apoptotic index of up to 91 percent, which persisted even 4 weeks following the initial injury, along with decreased muscle pathology and cellular apoptosis [108]. The same group also found that myotubes subjected to hypoxia for up to 5 h displayed up to 75 percent less apoptosis when treated with 1–100 µM NaHS, suggesting that H_2_S could be used to increase the time until critical ischemia begins [109]. There was no significant difference between apoptotic indexes using 1, 10, or 100 µM NaHS, suggesting that even low NaHS doses prevent hypoxia induced apoptosis [109]. The same study also demonstrated that mice treated with 10 µM NaHS prior to IR displayed less muscle pathology and apoptosis detected by TUNEL assay. The time of NaHS administration was critical, as only the mice treated with NaHS 20 min prior to reperfusion displayed less muscle pathology and apoptotic index. There were no significant differences in mice treated with NaHS 1 min prior to reperfusion [109], indicating that in spite of a short in vivo half-life H_2_S requires little time to act on target tissues. Indeed, it has been suggested that that H_2_S administration 1 h prior to reperfusion results in superior protection against IR and increased inhibition of JNK and NF-κB activation in kidney [110]. It has also been shown that IR increases mitochondrial superoxide and mPTP opening, leading to skeletal muscle apoptosis [111, 112]. It is plausible that as a free radical scavenger H_2_S can prevent apoptosis by preventing stress induced mPTP opening, in addition to the previously described activation of PKC [113]. Further work is needed to deduce the exact mechanisms of H_2_S in reducing IR associated stress.

## H_2_S and microRNAs associated with IR

In addition to activating Akt and inhibiting inflammation, H_2_S has been implicated in preventing apoptosis by attenuating microRNAs (miRNA): small endogenous non-protein coding RNA strands that act to regulate specific gene expression post-transcriptionally. Kang et al. [114]. pre-treated neonatal rat cardiomyocytes with 30 µM NaHS for 30 min prior to subjecting the cells to hypoxia for 24 h, followed by 2 h of normoxia. Along with increased Bcl-2 expression, and reduced apoptosis and lactate dehydrogenase (LDH) release following NaHS treatment, they also observed that IR increased expression of miRNA-1 (an inhibitor of Bcl-2) in cardiomycoytes, which was reduced by NaHS [114]. Mice subjected to peritonitis by injection of zymosan A that the H_2_S donor sodium sulfate (Na_2_S) reduced cardiac inflammation, apoptosis, and necrosis, and reduced myocardial infarct size by 63 percent, which correlated with increased presence of cardioprotective miRNA-21 [115]. Cells treated with the miRNA inhibitor antagomiR-21 did not display reduced inflammation and apoptosis when treated with Na_2_S, nor did miRNA-21 knockout mice [115], indicating that miRNA-21 is critical for regulating the anti-apoptotic effects of H_2_S. At least in cardiomyocytes, it appears that H_2_S reduces inhibitory miRNA-1 and induces miRNA-21, indicating that both pro-and anti-apoptotic miRNAs are potential targets for H_2_S. It is currently unknown if these or other miRNAs are affected in other muscle types. miRNAs are also implicated in apoptosis in non-muscle tissue that are H_2_S responsive. GYY4137 reduced TNFα induced apoptosis in spinal cord neurons by enhancing expression of miRNA-485-5p [116], suggesting that a variety of miRNAs involved in stress signaling an cell death can be modulated by H_2_S. We are likely only beginning to understand the extent of transcriptional regulation of genes by H_2_S and other gasotransmitters.

Many of the apoptotic regulators affected by H_2_S, and the cell types they have been identified in, are summarized in Table 1. H_2_S can regulate apoptosis in many cell types by regulating the Akt-JNK-Bcl-2 pathway, inhibiting mPTP opening, preserving mitochondrial integrity, reducing inflammation, and affecting miRNA expression. It remains to be deduced if these mechanisms are cell type specific, or ubiquitous across many IR types. Future research will identify other anti-apoptotic targets for H_2_S, and their roles in attenuation of IR in various tissues.

**Table 1:** Apoptotic regulators associated with ischemic-reperfusion injury that are responsive to hydrogen sulfide

H_2_S target	Cell/tissue type	Functions	References
Akt	Cardiac, renal, neural, hepatic	JNK, mTOR, GSK3β, NR2A and B activation	[32, 42, 93, 101, 117]
HSP	Retinal, hepatic	Thioredoxin-1 activation, ROS scavenging, reducing inflammatory cytokine induction	[93, 94, 102]
JNK	Cardiac, retinal, renal, hepatic, epithelial	Bcl-2 inactivation, cytochrome C release	[90, 94, 118, 119]
Beclin-1	Cardiac, hepatic	Decrease autophagosome formation	[39, 99]
GSK3β	Cardiac, hepatic, neural	Activate Bax, decrease LC3 and Beclin-1, inhibit mPTP opening,	[6, 72, 95, 101]
Bcl-2	Cardiac, renal, hepatic, neural, epithelial	Prevent cytochrome C release, inactivate Bax	[72, 91, 95, 99, 101, 102, 105]
TNFα, IL-6, IL-1β	Cardiac, skeletal muscle, lung	ROS increase, mPTP opening, Bax activation	[24, 99, 106–109, 118]
miRNA (1, 21, 485-5p)	Cardiac, neural	Reduce LDH, regulate transcriptional activation, reduce TNFα activity	[114–116]

## Clinical administration of H_2_S in IR

Despite the extensive research that has been conducted using H_2_S on rodent studies, to date there have been no human studies testing the efficacy of H_2_S donors on reducing IR. H_2_S can be released by fast or slow donors. NaHS is a fast release donor of H_2_S that exerts quick systemic effects. There is some evidence that slow release H_2_S donors such as diallyl trisulfide (DATS) and GYY4137 might exert longer lasting effects on Akt, eNOS, and prevention of cardiac ischemia [37, 43, 120]. This action might be further improved by combining slow release donors with nanoparticles that aid delivery have greater efficacy than donor alone, such as in using DATS combined with mesporous silica nanoparticles to enhance delivery of H_2_S to IR affected cardiomyocytes [37]. Patients experiencing IR that are able to quickly get to medical facilities might benefit from low doses of inhaled H_2_S can induce a hypometabolic state that limits tissue oxygen utilization and prevents further organ damage [86, 87]. Although hydrogen sulfide in air has a longer half-life than in solution (up to 3 days), care must be taken when administering gaseous hydrogen sulfide, and this method is not practical for immediate application of H_2_S in acute injury. Injectable hydrogen sulfide, likely using slow release donors, will be necessary for these applications. However, since H_2_S has a short half-life in vivo and solutions (about 12 min), it will be necessary to quickly prepare and administer donors. Tissue specific targeting of H_2_S is needed to prevent the compound from altering function in undamaged tissue, and travel only to affected targets such as IR affected muscle. It might also be beneficial to use drugs that increase levels of endogenous H_2_S synthesis by increasing activities of the three main H_2_S producing enzymes (CBS, CSE, 3-MST). To our knowledge there are not yet any drugs that would increase endogenous expression of these enzymes, although ingestion of foods high in H_2_S such as garlic might result in increased endogenous levels [121]. Future research is needed to develop methods to efficiently deliver H_2_S to target tissues while taking care to avoid overdosing patients, which can lead to hydrogen sulfide poisoning and associated complications.

## Conclusions and future perspectives

H_2_S has been demonstrated in many studies to improve response to IR by affecting various signaling pathways involved in metabolic signaling (particularly Akt), mitochondrial integrity and ETC activity, apoptosis, and cell survival in many tissue types. The nature of the H_2_S donor might be important for determining its effectiveness. Several studies have shown that the slow release donors such as GYY4137 and diallyl trisulfide that dissolve slower in solution may be more effective in reducing apoptosis and inflammation than fast release donors such as NaHS [37, 106, 119]. It has been suggested that H_2_S might work best when combined with other therapies. A widely used therapy for limb and organ transplantation is hypothermia, which can cause damage to the tissues. Several studies have shown that a combination of H_2_S and mild hypothermia can prevent IR and limb infection following severe injury, along with extending organ life and reducing tissue metabolic activity when used in transplants [14, 89, 92, 122–126]. The major effect of combination therapy is likely ROS scavenging, as therapies with other ROS scavengers such as ozone have proven effective in reducing skeletal muscle IR when combined with hypothermia [16]. Further mechanisms that are involved in H_2_S-hypothermia limb and organ preservation, such as metabolic pathway regulation, should be deduced in future studies.

In conclusion H_2_S is a multifaceted regulator of cell signaling that acts on many pathways and tissue types affected by IR. Future research will determine further regulatory mechanisms affected by this gasotransmitter, its effects on post-transcriptional regulation in different cell types, the effectiveness of combined therapies to reduce IR complications, and the possibility of using H_2_S to extend the critical ischemic time prior to reperfusion in order to allow better treatment of limb and tissue damage in severe injuries.

## Abbreviations

3-MST: mercaptopyruvate sulphur transferase
AMPK: adenosine monophosphate kinase
Bcl: B cell lymphoma
cAMP: cyclic AMP
CBS: cystathionine β-synthase
CO: carbon monoxide
CSE: cystathionine γ-lyase
eNOS: endothelial nitric oxide synthase
ETC: electron transport chain
FADH_2_: flavin adenine dinucleotide
FoxO: forkhead box
GSK: glycogen synthase kinase
GYY4137: Morpholin-4-ium 4 methoxyphenyl (morpholino) phosphinodithioate
HSP: heat shock protein
IGF: insulin growth factor
IL: interleukin
IR: ischemia-reperfusion injury
H_2_S: hydrogen sulfide
JNK: c-Jun-N-terminal kinase
LC3: microtubule-associated protein 1A/1B-light chain 3
LDH: lactate dehydrogenase
miRNA: micro RNA
MnSOD: magnesium superoxide dismutase
mPTP: mitochondrial permeability transition pore
MPTP: 1-methyl-4-phenyl-1,2,3,6-tetrahydropyridine
mTOR: mammalian target of rapamycin
Na_2_S: sodium sulfate
NADH: nicotinamide adenine dinucleotide
NAC: N-acetyl-l-cysteine
NaHS: sodium hydrosulfate
NO: nitric oxide
NR: glutamate NMDA receptor subunit epsilon
PDE2A: phosphodiesterase 2A
PI3 K: phosphoinositol-3 kinase
ROS: reactive oxygen species
SUR: sulfonylurea receptor
TNFα: tumor necrosis factor α
UCP: uncoupling protein
VEGF: vascular endothelial growth factor
